# Our Initial Experience of Left Bundle Branch Area Pacing (LBBAP) With Stylet-Driven Pacing Leads (SDL) in a Tertiary Care Center

**DOI:** 10.7759/cureus.84845

**Published:** 2025-05-26

**Authors:** Goutam Kumar, Chandrabhanu Chandan, Nithyapriya T K, Ravi V Prasad

**Affiliations:** 1 Department of Cardiology, Indira Gandhi Institute of Medical Sciences, Patna, IND

**Keywords:** conduction system pacing, left bundle branch area pacing, physiologic pacing, stylet-driven pacing leads, tertiary care center

## Abstract

Background

The use of left bundle branch area pacing (LBBAP) as a substitute for conduction system pacing (CSP) is becoming increasingly popular. Thus, this study was performed to evaluate the feasibility, safety, and outcomes of LBBAP with stylet-driven pacing leads (SDL) in a tertiary care center in Eastern India.

Materials and methods

This single-center, retrospective study was conducted at the Department of Cardiology, Indira Gandhi Institute of Medical Sciences (IGIMS), Patna, Bihar, India. The study included data from 20 patients over one year, i.e., from December 2023 to December 2024. The procedure-related characteristics included fluoroscopy exposure duration, doses, and patterns of electrocardiograms (ECG) and intracardiac electrograms (EGM) that were documented during implantation. The Institutional Ethics Committee (IEC), IGIMS, Patna, Bihar, India, has granted ethical approval (approval no. 291/IEC/IGIMS/2025, dated January 9, 2025).

Results

Implant success was observed in 18 (90%) patients. Significant differences were observed in bundle branch block and pacing indications with p-values <0.001 and 0.002, respectively. It was noted that a highly statistically significant result was obtained when unipolar impedance and bipolar impedance were compared, with a p-value of 0.002.

Conclusion

Our study found that permanent LBBAP is a viable physiological pacing method with a 90% success rate. Moreover, septal perforation complications were not noted. Among the limitations of the study were the small number of patients, which might affect the efficiency of the results, and the shorter duration of time. Additionally, the study was retrospective; thus, similar records of some parameters were difficult to find.

## Introduction

The use of left bundle branch area pacing (LBBAP) as a substitute for conduction system pacing (CSP) is becoming increasingly popular. It is a novel strategy to keep pace with near-normal synchrony, shorting out limitations of conventional right ventricle (RV) pacing of the apical. The pacing of the conventional RV has many undesirable consequences, such as increased incidence of heart failure, hospitalization because of heart failure, and development of atrial arrhythmias.

There are limitations of cardiac resynchronization therapy (CRT) as well, i.e., a high percentage of non-responders, anatomical abnormality to place the left ventricle (LV) lead, and a time-consuming procedure, which prompted the development of a more physiological novel pacing strategy. Thus, LBBAP emerges as a new armamentarium in the pacing field, overcoming the limitations of His Bundle pacing as well. In the first-in-human study conducted in 2016, it was shown that persistent LV septal pacing (LVSP) along with the pathway of transseptal ventricular transseptal was found to be feasible [1].

Later, Huang et al. refined this method and showed that the transseptal approach could be used to directly pace the proximal left bundle branch pacing (LBBP) [2]. However, results of many studies have reported the feasibility and safety of the LBBAP procedure instead of conventional RV pacing and bilateral CRT [3-5]. Typically, a pacing lead without a lumen of helical design was used for LBBAP [6]. There are now comprehensive operator manuals for LBBAP utilizing lumen-less leads (LLL) available [7]. Recent reports show that LBBAP with conventional stylet-driven pacing leads (SDL) is safe and practical [8,9].

LBBAP includes both LVSP, which activates the left septal endocardium without first activating the left bundle branch (LBB), and LBBP, which activates the left cardiac conduction system (CCS) either selectively or non-selectively [10]. With improved stiffness that facilitates septal penetration, the use of SDLs in LBBAP has been enforced. These leads also enable continuous pacing through the stylet, which, in contrast to the interrupted technique used in LLLs, permits accurate tracking of variations in QRS form and pacing impedance while holding the lead [11,12]. Until now, very few studies have been observed to perform the procedure of LBBAP along with the use of SDLs.

The study was performed to evaluate the feasibility, safety, and outcomes of LBBAP with SDL in a tertiary care center in Eastern India, prompted by a lack of data on the safety and efficacy of LBBAP with SDL in Indian patients.

## Materials and methods

Study design

A single-center, retrospective study was conducted at the Department of Cardiology, Indira Gandhi Institute of Medical Sciences (IGIMS), Patna, Bihar, India. The study included data from one year, i.e., from December 2023 to December 2024.

Study population

Overall, the study considered data from 20 patients who underwent LBBAP with SDL at our institution. The inclusion criteria involved all adult patients over 18 years of age undergoing LBBAP procedures with the use of SDL. The participants who were excluded were those aged less than 18 and who had undergone LBBAP procedures using other pacing leads.

Data collection and study procedure

At enrollment, all patients' medical histories and demographic information were gathered. Procedure-related characteristics included fluoroscopy exposure duration, doses, and patterns of electrocardiograms (ECG) and intracardiac electrograms (EGM) that were documented during implantation and after implantation for defining procedural success by observing these specific characteristics. Following implantation, pacing characteristics such as the amplitude of the R wave, impedance of pacing, and threshold of pacing were measured. Helix entrapment in the septum, pneumothorax, hemothorax, and cardiomyopathy were among the significant acute procedure-related adverse events for which safety endpoint data were gathered, mainly lead-related complications or procedural issues. The equipment that was considered included Siemens Artis zee (Siemens Healthineers, Germany) for fluoroscopy; other than this, intracardiac EGMs were recorded using an EP recording system, e.g., EP-WorkMate™ (Abbott Laboratories, USA), and pacing was done using a stimulator.

Using a customized delivery sheath, which was facilitated through a preshaped sheath (Selectra 3D, Biotronik SE & Co., Berlin, Germany), the 5.6F SDL with extendable helix (Solia S60, Biotronik SE & Co.) was implanted deep into the septum and around 1.5-2 cm distal to the His Bundle in order to accomplish LBBAP. Electrophysiological parameters, complications, and implant success rates were evaluated.

The first line of therapy that was considered in the study was LBBAP. Clockwise turn of the sheath, advance it into the RV, followed by an anticlockwise turn, and the sheath is placed perpendicular. It is recommended to rotate the lead rapidly.

At this point, the current of injury (COI) was noted, which was shifted along with the position of the lead. The W pattern in the paced QRS was first shifted to the end of the QRS from the nadir in the lead V1 notch at the nadir. An increase in unipolar pacing impedance, followed by a slight fall, but not below 500 mV, was then recorded.

**Figure 1:**
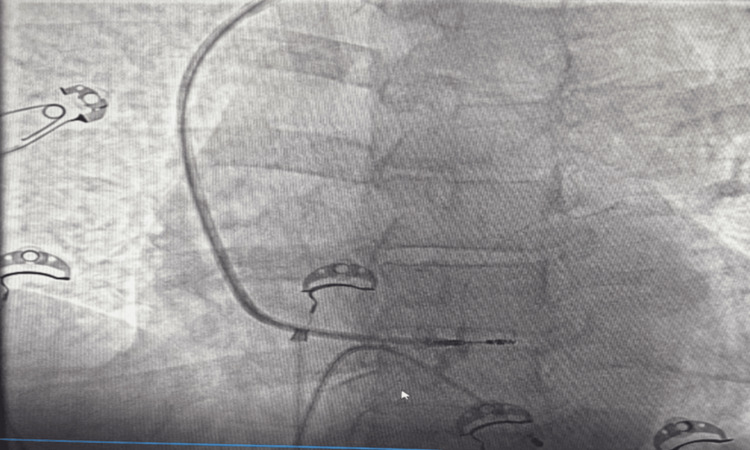
LBBAP procedure done using stylet-driven pacing leads (SDL) LBBAP: left bundle branch area pacing

Purkinje potentials were noted. Pacing thresholds were determined by analyzing the change in capture of selective and nonselective LBB. These events were typically seen at near-threshold pacing outputs. The fast revolutions are then repeated after the lead is released. The EGM would frequently record the myocardial COI. Figure 1 represents the procedure of LBBAP using SDL.

Statistical analysis

Microsoft Excel (Microsoft Corporation, Redmond, Washington) was used for the initial data entry. For further analysis of data, IBM SPSS Statistics for Windows, Version 26 (Released 2019; IBM Corp., Armonk, New York) software was used. The data were displayed as either n (%) or mean±standard deviation (SD). A paired t-test was used to analyze dependent variables. For other categorical data, the chi-square goodness-of-fit test was employed. A p-value was considered significant if it was less than 0.05.

Ethical clearance

The Institutional Ethics Committee (IEC), Indira Gandhi Institute of Medical Sciences (IGIMS), Patna, Bihar, India, has granted ethical clearance with approval number 291/IEC/IGIMS/2025, dated January 9, 2025.

## Results

Table 1 represents baseline characteristics among participants. The participants were 70±11 years old on average, with 12 (60%) participants being female and eight (40%) participants being male. Three (15%) individuals had a right bundle branch block, whereas 16 (80%) had an LBB block. One (5%) participant had an intraventricular conduction defect. Significant differences were observed in bundle branch block and pacing indications with p-values <0.001 and 0.002, respectively.

**Table 1:** Baseline characteristics of the participants Data were presented as either mean±SD or n (%). The chi-square test was employed to obtain the p-value. A p-value was considered significant if it was less than 0.05. LVEF: left ventricular ejection fraction; TR: tricuspid regurgitation

Parameters	Value	Chi-square value	p-value
Age (in Years)	70±11	-	-
Female Participants	12 (60%)	0.8	0.37
Male Participants	08 (40%)		
Comorbidities			
Hypertension	15 (75%)	-	-
Atrial Fibrillation	12 (60%)		
Coronary Artery Disease	10 (50%)		
Diabetes Mellitus	13 (65%)		
Heart Failure	09 (45%)		
Previous Stroke	04 (20%)		
Echocardiography			
Baseline LVEF (in %)	52.3±8.7	-	-
LVEF <50%	04 (20%)		
Moderate-to-Severe TR	03 (15%)		
Bundle Branch Block			
Right Bundle Branch Block	03 (15%)	19.89	<0.001
Left Bundle Branch Block	16 (80%)		
Intraventricular Conduction Defect	01 (5%)		
Duration of QRS (ms)			
QRS Duration (>130 ms)	08 (40%)	0.8	0.37
QRS Duration (<130 ms)	12 (60%)		
Pacing Indications			
Sinus Node Dysfunction	04 (20%)	12.39	<0.002
Cardiac Resynchronization Therapy	02 (10%)		
Atrioventricular Block	14 (70%)		
Duration of Procedure (in mins)	90±30	-	-
Fluoroscopic Time (in mins)	15±8.2	-	-

Table 2 depicts the comparison of parameters among participants. Implant success was observed in 18 (90%) patients. Eight (40%) participants demonstrated the potential of LBB. The unipolar and bipolar pacing thresholds at 0.4 ms were found to be 0.8±0.5 V and 1.0±0.9 V, respectively. It was noted that a highly statistically significant result was obtained when unipolar impedance and bipolar impedance were compared, with a p-value of 0.002. The success rate of the implant was highly statistically significant, with a p-value less than 0.001.

**Table 2:** Comparison of parameters among participants Data were presented as either mean±SD or n (%). The chi-square test or paired t-test was used to obtain the p-value. A p-value was considered significant if it was less than 0.05. LBB: left bundle branch; LVAT: left ventricular activation time

Parameters	Value	Chi-square/t-statistic value	p-value
Procedural Implant Success	18 (90%)	12.8	<0.001
Failure of Implant	02 (10%)		
Presence of LBB Potential	08 (40%)	0.8	0.37
Absence of LBB Potential	12 (60%)		
Baseline QRS Duration (ms)	114±29.8	0.34	0.74
Paced QRS Duration (ms)	112±11.7		
Mean LVAT High Output	71.7±11	1.22	0.24
Mean LVAT Low Output	74.7±11		
Pacing Characteristics at Implant			
Unipolar Pacing Threshold at 0.4 ms, V	0.8±0.5	-0.19	0.84
Bipolar Pacing Threshold at 0.4 ms, V	1.0±0.9		
Unipolar R wave Amplitude (mV)	9±6	-0.56	0.58
Bipolar R wave Amplitude (mV)	10±5		
Unipolar Impedance (ohms)	489±109	3.30	0.002
Bipolar Impedance (ohms)	593±89		

Hemothorax was observed in one patient (5%). The major complication, helix entrapment in the septum, was seen in two (10%) patients. Table 3 shows the complications of LBBAP using SDL among the participants.

**Table 3:** Complications of LBBAP using SDL among the participants Data were presented as n (%). LBBAP: left bundle branch area pacing; SDL: stylet-driven pacing leads

Complications	Value
Pneumothorax	0 (0%)
Hemothorax	1 (5%)
Cardiomyopathy	0 (0%)
Septal Perforation	0 (0%)
Helix Entrapment in the Septum	2 (10%)

## Discussion

The current study was conducted to evaluate the safety, efficacy, and feasibility of LBBAP with SDL. The implementation of the LBBAP method in a tertiary facility is evidenced by the study's major findings, which also showed that numerous new implanters had acceptable success rates with low rates of acute procedural problems.

Our study's procedural length and QRS improvement were comparable to those seen in prior research [12,13]. The participants in our study were 70±11 years old on average. Byeon et al. conducted a similar trial in 2022 to report on their experience with LBBAP using new pre-shaped delivery sheaths and leads. According to the study, the participants were 71±16 years old [14]. In 2022, a different study by De Pooter et al. used SDL with an extended helix design to assess the safety and feasibility of LBBAP in a multicenter patient population. The study's mean participant age was 76 ± 39 years among 353 patients included [15].

Our study enrolled 12 (60%) female participants and eight (40%) male participants. In contrast, the study by De Pooter et al. in 2022 reported enrollment of 43% of female participants [15]. Another study by Kamsani et al., conducted to assess the acute procedural success and first learning experience of implementing this approach at an academic training center, reported the inclusion of 60.9% male participants [16].

The procedural length of our study was 90±30 minutes. A similar study by Kamsani et al. also reported a procedural time of 74.1±23.5 minutes [16].

Implant success has been observed in 18 (90%) participants in our study. A study by De Pooter et al. in 2022 also showed a 90% success rate of implants [15]. Byeon et al.'s study found that 83% of patients responded well to LBBAP [14].

A study done by Ponnusamy et al. in 2021 to evaluate the mid-term results, electrophysiological parameters, practicality, and effectiveness of LBBP in the Indian population concluded that with a 94% acute success rate, LBBP is a safe and efficient physiological pacing technique. The pacing settings did not change during the 12-month follow-up period [17].

Another study, done by Su et al. in 2021 to assess the safety and viability of LBBP in a sizable, diverse patient cohort with ongoing monitoring, suggested that LBBP is doable with excellent success rates and minimal complications during long-term follow-up. Thus, for patients with a bradycardia or heart failure pacing indication, LBBP seems to be a dependable physiological pacing technique [6].

In our study, the only major complication was helix entrapment in the septum that was observed in two (10%) patients. In contrast, the majority of cases in other investigations have shown numerous additional problems, including pneumothorax, hemothorax, septal perforation, and septal coronary artery fistula [14,15]. Other than this, a study by Kamsani et al. reported septal perforation in seven (10.1%) participants and damaged lead helix requiring a second lead in three (4.3%) participants [16].

Because prior research has largely concentrated on a small number of clinics and implanters, the question of whether LBBAP is useful in real-world practice has emerged. Our study makes a substantial contribution to this field by illuminating the viability and usefulness of introducing LBBAP in a low-to-medium volume training facility, especially for novice implanters.

Limitations

Among the limitations of the study were the small number of patients, which might affect the efficiency of the results, and the shorter duration of time. Additionally, the study was retrospective; thus, similar records of some parameters were difficult to find. The lack of a control group may also not help in reflecting real-world effectiveness. Long-term follow-up data were not considered for patients with complications, which might influence our study results. Further, such studies must look for long-term follow-up data among such participants.

## Conclusions

Our study found that permanent LBBAP is a viable physiological pacing method with a 90% success rate. Moreover, no complications of septal perforation were noted. Additionally, LBBAP has gained significant pace in recent years. Thus, it can be said that LBBAP can be safely used with SDL, which could encourage more people to use it as a novel pacing technique. Further prospective studies with larger populations might help ensure the efficacy and safety of the LBBAP procedure, along with the use of SDL.
